# Air quality changes in Taiwan over the past decades and during the COVID-19 crisis

**DOI:** 10.1007/s44195-023-00036-4

**Published:** 2023-04-03

**Authors:** Chih-wen Hung, Ming-Fu Shih

**Affiliations:** Department of Geography, National Taiwan Normal University, 162, Sec 1, Hoping East Rd, 106 Taipei, Taiwan

**Keywords:** Air quality, Air pollutants, COVID-19, Taiwan

## Abstract

Over the past decades, Taiwan has achieved remarkable goals in air pollution reduction with the concentrations of several common air pollutants such as CO, NO_x_, PM_10_, PM_2.5_, and SO_2_ going down. In contrast to these achievements, the mitigation of O_3_ remains extremely tough due to the complexity of its formation process involving synergistic effects of precursor reductions and meteorological influences. During the local COVID-19 crises in Taiwan and the Level 3 alert in 2021, air pollutants directly emitted from the traffic such as CO and NO_x_ present clear relationships with the drop of the recorded freeway traffic volume due to the alert, while PM_10_ and PM_2.5_ which are also relevant to the traffic do not show indications of being greatly influenced by the decrease of the traffic flow. Although road traffic is not regarded as a main source of SO_2_ by current understanding, the unusual SO_2_ variation patterns found in this study suggest a prolonged impact for months from the changes of travel behavior during the epidemic. In contrast, the epidemic did not exert influences on industrial SO_2_ concentration which accounts for a large portion of total SO_2_ in Taiwan, and a similar scenario is also seen in each type of O_3_ monitoring. Although some results discussed in this study are not in line with current consensuses and understandings in terms of the nation of certain air pollutants, these findings may disclose new perspectives which could be a potential benefit to air quality improvement projects in the future.

## Introduction

Air pollution-related issues are in crisis for most countries to seriously face up and Taiwan cannot stay out of the way. When organisms are exposed to high concentrations of air pollutants for a long time, the risk of causing serious respiratory diseases will greatly increase, which heavily aggravates the social burden. Local sources of air pollution such as industries and automobiles (Wu et al. 1998; Li et al. 2018) account for the majority of polluted emissions in Taiwan, and overseas air pollutants, most of them coming from China (Chen et al. 2014), further worsen air quality (Chou et al. 2006; Chiang et al. 2009; Zhang et al. 2015). As a result, Taiwan often fails to meet domestic air quality standards.

Several air quality indicators have been used in order to provide a straightforward way for the public to understand current air quality conditions. In July 1993, the Pollution Standards Index (hereafter PSI) was introduced by the Environmental Protection Administration, Executive Yuan (hereafter EPA) to become the indicator for air quality monitoring in various regions of Taiwan (Lin et al. 2003; Jan 2015). However, significant diversities between the PSI results and actual air quality frequently occurred since the operation of the PSI. As a new indicator to replace the discontinued PSI, the Air Quality Index (hereafter AQI) featuring stricter standards and more monitoring air pollutants was adopted since September 2016 (Yu 2018). The PSI and AQI calculate the concentration of individual air pollutants as sub-indicators and then applied the most severe one as the final index value for a day, and the unhealthy air quality condition is defined when the index value is higher than 100 (EPA 2015).

Previous research and the latest report from Taiwan Emission Data System (hereafter TEDS) pointed out that dust from road traffic and the development of mountainous areas or river banks, traffic emissions and industrial particulate pollutants contribute to the majority of PM_10_ in Taiwan (Zhang et al. 2015; EPA 2021a). Among total particulate matter emissions, PM_2.5_ accounts for a large portion (Chuang et al. 2008) and mainly comes from traffic emissions and the steel industry depending on seasons (Hwang et al. 2018). In addition to the particulate matter, according to the TEDS, both industries (including power plants) and road traffic contribute to large portions of total NO_x_ emissions, and the majority of SO_2_ emissions come from industries and power plants (EPA 2021a). Given that NO_x_ and SO_2_ are the precursors of PM_2.5_ and acid rain, and NO_x_ is related to the formation of O_3_, many experts have laid great stress on controlling NO_x_ and SO_2_ emissions from the industry and the traffic. However, air quality management is not just a domestic affair. During boreal winter and spring, air pollutants emitted from China would be transported by the winter monsoon to Northern Taiwan (Lin et al. 2005) and sequentially raise the concentrations of local SO_2_, PM_2.5_ and O_3_ through dust storms and haze events (Cheng et al. 2008). Therefore, air pollution reduction plans for secondary air pollutants such as PM_2.5_ and O_3_ are always exceptionally challenging.

The recent COVID-19 epidemic was firstly identified in Hubei Province of the People’s Republic of China at the end of 2019 (Velavan and Meyer 2020) and since then the epidemic has been rapidly spreading worldwide. In response, many countries in succession closed the borders or put on mandatory lockdowns to combat domestic outbreak. By early April 2020, over a third of the global population was involved in some forms of movement restriction or lockdown (Koh 2020). Unprecedented upheaval in travel behavior due to the closure of borders and lockdowns became a serious issue for the world to face, and the cancellations of international travelling between most of the countries led to a big fall in flights and passenger loads, causing a heavy blow to the aviation sector (Sun et al. 2020; Andreana et al. 2021; Bao et al. 2021).

Although the sudden pause of industries, transportations and activities brought about shutdown of satisfying lifestyle that people used to, the accompanying unprecedented decrease of polluted emissions also provided an unintended and temporal breathing space for the environment. The comprehensive cancellations of flights could significantly impact on the reduction in greenhouse gases and other air pollutants (Ngo et al. 2022). Aside from the reduction of aviation emissions, lockdowns and other measures implemented for the epidemic also caused great effects to air quality. Barua and Nath (2021) studied CO emissions globally during a period when most countries went into strict lockdowns and suggested that longer term lockdowns and mobility restrictions slowed down CO emissions. Gao et al. (2021) found a relationship between the lockdown and the reduction in the concentrations of most air pollutants in China and pointed out that the lockdown only temporarily reduced the air pollution. Steinbrecht et al. (2021) indicated that COVID-19-related emissions reductions were the major cause for the observed unusually reduced free tropospheric O_3_ in northern extratropic throughout the spring and summer in 2020. Besides, certain industry could also be benefited from the decrease of emissions. During the lockdowns in 2020, the declining seasonal O_3_ concentration and positive impacts on crop yields could be a consequence of the unintended decrease of emissions (Dentener et al. 2020).

It seems reasonable to expect less polluted air during the periods of lockdowns; however, it is not always the case in some places where higher concentrations of certain air pollutants could be observed. Shakoor et al. (2020) found that the concentrations of various air pollutants decreased in the studied provinces of China during the lockdown, while air quality only partially improved in the studied states of the USA during the lockdowns with the concentrations of certain air pollutants going up. In some cases, Kerimray et al. (2020) reported higher concentrations of secondary pollutants and other pollutants such as benzene and toluene during the lockdowns. Similar results were proposed by Huang et al. (2021) who indicated that large decreases in certain air pollutants such as NO_x_ during lockdowns could facilitate the formation of secondary particulate matter. Furthermore, Tang et al. (2021) found that O_3_ had regional disparities in the changes of emissions with a rise in most areas of East Asia and Europe but a non-negligible declining signal in North America. These researches implied the complexity for the mitigation of secondary pollutants which should take synergistic effects of precursor reductions and meteorological influences into consideration.

In late April 2021, Taiwan for the first time encountered a major impact of the epidemic with hundreds identified local cases and lives lost every day since the first outbreak of COVID-19 in late 2019. To restrain the spread of the virus, the nationwide Level 3 alert was announced by the Central Epidemic Command Center (CECC) in May 2021, which led to sudden pause for bustling anthropogenic activities. Given that the impact of COVID-19 on air pollution has been widely studied at different locations across the world, the aims of this study set to not only review the effects of air quality improvements over the past decades, but also give an overview of the impact of COVID-19 on air quality specifically for Taiwan. Hence, this study examines the concentrations of various major air pollutants in 1994–2021 including a period covering both pre-COVID-19 years and epidemic years to evaluate the impact of the epidemic on local air quality changes in Taiwan. The relevant results provide an overview regarding temporal changes and regional differences of these air pollutants recorded by different types of air quality stations. The information of the data used for the study will present in Sect. 2. The changes of air quality through different temporal perspectives and the impact of COVID-19 on air quality will be investigated and discussed in Sects. 3 and 4. Finally, Sect. 5 briefly goes over the work of the study.

## Data

The establishment of the first generation of air quality monitoring network in Taiwan began in 1982 for the purpose of providing accurate and detailed air pollutant information. The initial framework of the monitoring network was formed in September 1993 and since then air quality monitoring data have been recording in each city and county. The equipment and the monitoring network have been developing and expanding over the years to improve the accuracy and variety of monitoring items. Now the monitoring network has been upgraded to its third generation with a variety of tasks. Among these monitoring stations, the traditional air quality monitoring stations are distributed across all counties and cities in Taiwan island, Penghu, Kinmen and Matsu, and can be further subdivided into numerous types by their monitoring purposes: the general air quality monitoring station (hereafter general station) for air quality monitoring in populous areas, industrial area air quality monitoring station (hereafter industrial station) for air quality monitoring in industrial areas, traffic area air quality monitoring station (hereafter traffic station) for air quality monitoring in areas with heavy traffic, national park air quality monitoring station (national park station) for understanding the current states and future trends of air quality in reserved areas, background air quality monitoring station (background station) for large-scale air quality or transboundary pollutants monitoring, and other air quality monitoring station (other station) for special purposes.

Considering the comprehensive air quality monitoring project began in September 1993, this study obtained air quality monitoring daily data within each complete monitoring year during 1994–2021 through the Environmental Protection Administration Environmental Information Open Platform at https://data.epa.gov.tw/. This study focuses on several common air pollutants such as CO, NO_x_, SO_2_, PM_10_, PM_2.5_ and O_3_ monitored by 60 general, 6 traffic and 5 industrial stations together with two air quality indicators, the PSI and AQI. Among all the air quality monitoring stations on the island of Taiwan, the general stations account for the vast majority and most of them gather in Northern, Central and Southern Taiwan where the three major metropolitan areas (Taipei, Taichung and Kaohsiung) locate for the purpose of monitoring air quality in populous environments. Most of the traffic stations gather in Taipei and Kaohsiung metropolitan areas with high traffic volume and the industrial stations disperse near the major industrial districts in several counties of Central Taiwan. All these materials allow our analysis to explore temporal and spatial characteristics of air pollutants in different regions of Taiwan, especially during the COVID-19 crisis with a shorter 5-year studied period in 2017–2021 covering years before and after the outbreak of the epidemic.

Traffic statistics of various types of vehicles (M03A) was also obtained through Traffic Data Collection System (TDCS) managed by the Freeway Bureau of the Ministry of Transportation and Communications (MOTC) at https://tisvcloud.freeway.gov.tw/history/TDCS/M03A/. The data collects traffic flow information on the north–south direction freeways (national freeways 1, 3, 5 and 3A) at an interval of 5 min through the Electronic Toll Collection (hereafter ETC) with total amount of 335 segments to reflect variations of the intercity traffic volume on a daily basis. To examine the changes in travel behavior during COVID-19 and the subsequent Level 3 alert in Taiwan, a same 5-year period (2017–2021) for the freeway traffic record data was obtained.

## Air quality changes in Taiwan over the past decades

### The incidence of the major air pollutants in severe air quality conditions by the PSI and AQI

**Fig. 1:**
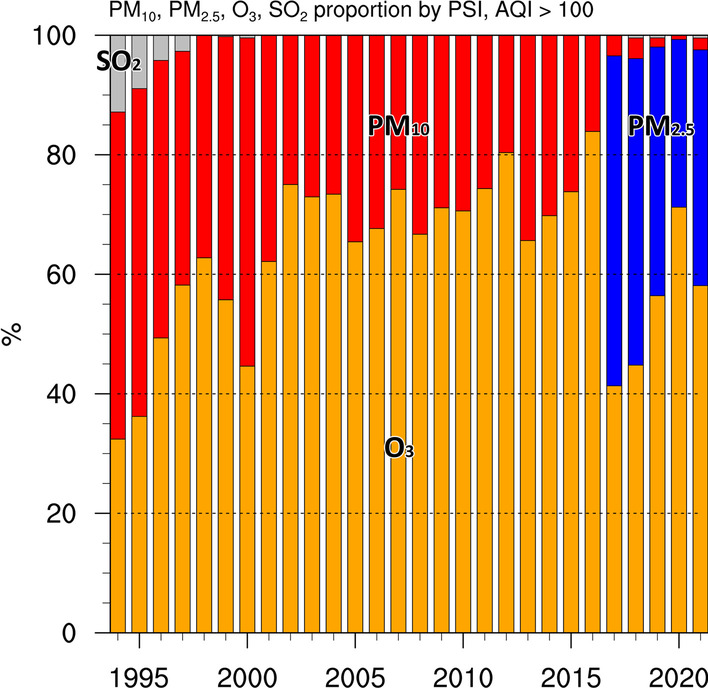
The incidence of the major air pollutants in severe air quality conditions defined by the PSI and AQI over 100. PM_10_, PM_2.5_, O_3_ and SO_2_ are shown in red, blue, orange and gray

The former air quality indicator PSI and the current AQI calculate the concentrations of individual air pollutants as sub-indicators, including CO, NO_2_, SO_2_, PM_10_ and O_3_ for the PSI and additional PM_2.5_ and the O_3_ maximum 8-h average value for the AQI. Among these sub-indicators, the most severe one is chosen as the major air pollutant and the associated value is regarded as the final index for an air quality monitoring station on a day. Considering that the PSI and AQI in Taiwan were respectively introduced in July 1993 and in September 2016, this study examines all available air quality monitoring station in each complete monitoring year (1993 for the PSI and 2016 for the AQI are ignored because of incomplete monitoring) and calculates the incidence for each major air pollutant during severe air quality conditions (the PSI or AQI value over 100), shown in Fig. 1.

Through the result in Fig. 1, among all the sub-indicators in the PSI system, O_3_, PM_10_ and SO_2_ almost dominate the major air pollutants each year during the period of 1994 to 2016, while the importance of SO_2_ gradually decreases and it almost withdraws from the group of the major air pollutants since 1998. For the rest of the years until 2016, O_3_ and PM_10_ almost entirely dominate the statistics every year with O_3_ quickly gaining its impact before 2002 and then gradually aggrandizing itself until 2016.

Based on the air pollutants considered for the PSI, PM_2.5_ and the O_3_ maximum 8-h average value were additionally adopted for the calculation of the AQI system when this new indicator was launched in 2016. Since 2017, the first complete AQI monitoring year, PM_2.5_ joins the group of the major air pollutants along with the other two long-time dominators, while PM_10_ becomes the least important one compared with the others. The fact that PM_2.5_ becomes one of the major air pollutants stresses the immediate need of reducing particulate matter emissions in an air quality improvement plan. In addition, it also shows that people could have seriously underestimated the threat of particulate matter particularly small particles during the years when the PSI was operated (Fig. 1). Besides, since particulate matter gives way to O_3_ in recent years from the contribution point of view, it sends a clear message for Taiwan that the society should pay more attention on the management of O_3_ concentration when drawing up a new plan for further air quality improvement in the future. In fact, plans for the mitigation of O_3_ are always troublesome. Although controlling the emission of precursors such as NO_x_ and Volatile Organic Compounds (hereafter VOCs) is theoretically effective, the formation mechanism of O_3_ is rather complicated since NO_x_ acts as a precursor and restrainer in the formation process of O_3_. In that regard, the formation of O_3_ is not only subject to the concentrations of VOCs but the prevailing ratio between VOCs and NO_x_, which implies that the excessive suppression of NO_x_ emissions could lead to a rise in O_3_ concentration (Sillman 1999).

### Interannual variations of the major air pollutants

**Fig. 2:**
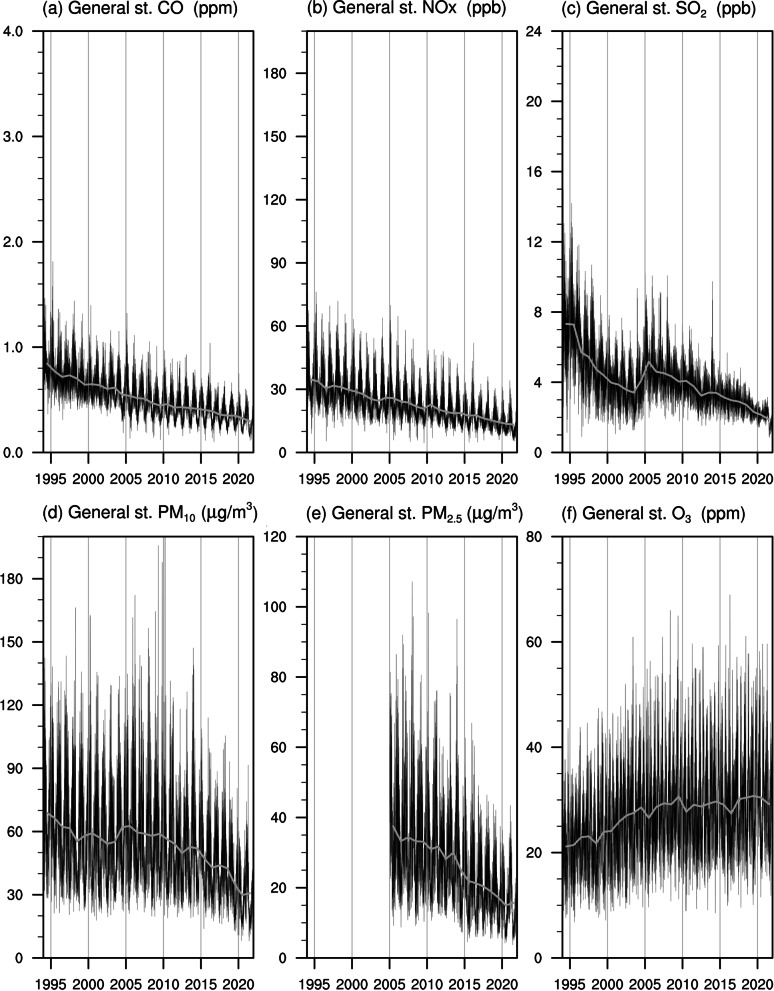
Variations of daily (**a)** CO, (**b)** NO_x_, (**c)** SO_2_, (**d)** PM_10_, (**e)** PM_2.5_, (**f)** O_3_ concentration (black line) monitored by the general stations with annual mean value for each year (gray line)

In order to gain an insight into the achievements that Taiwan made in terms of air quality improvement, this study sorts all the air quality monitoring stations by type and presents the concentration of each major air pollutant on both daily and yearly bases in 1994–2021. The results of the air pollutants observed by all the general stations across Taiwan are presented in Fig. 2. Almost all kinds of air pollutants present a rather steady decline in concentration over the analyzed years although there are some temporal rebounds seen in the concentrations of SO_2_ and PM_10_ in 2003–2005. The only exception goes to the concentration of O_3_ with a clear increase before 2005, while the rate of increase becomes moderate after 2005 and the concentration stays relatively stable afterwards. Although the result of O_3_ mitigation is not as promising as other air pollutants, the slowing growth of O_3_ concentration after 2005 still reveals a positive signal of the measures implemented. Note that some extremely high values of PM_10_ concentration (outside the scale of the plot) were recorded around 2010, which was a consequence of the iconic severe dust storm event occurring in March 2010. Since smaller particles are usually inefficient to be transported in such long distance all the way from China, it could be the answer about why similar extreme values at the same time were not recorded in PM_2.5_ monitoring.

**Fig. 3:**
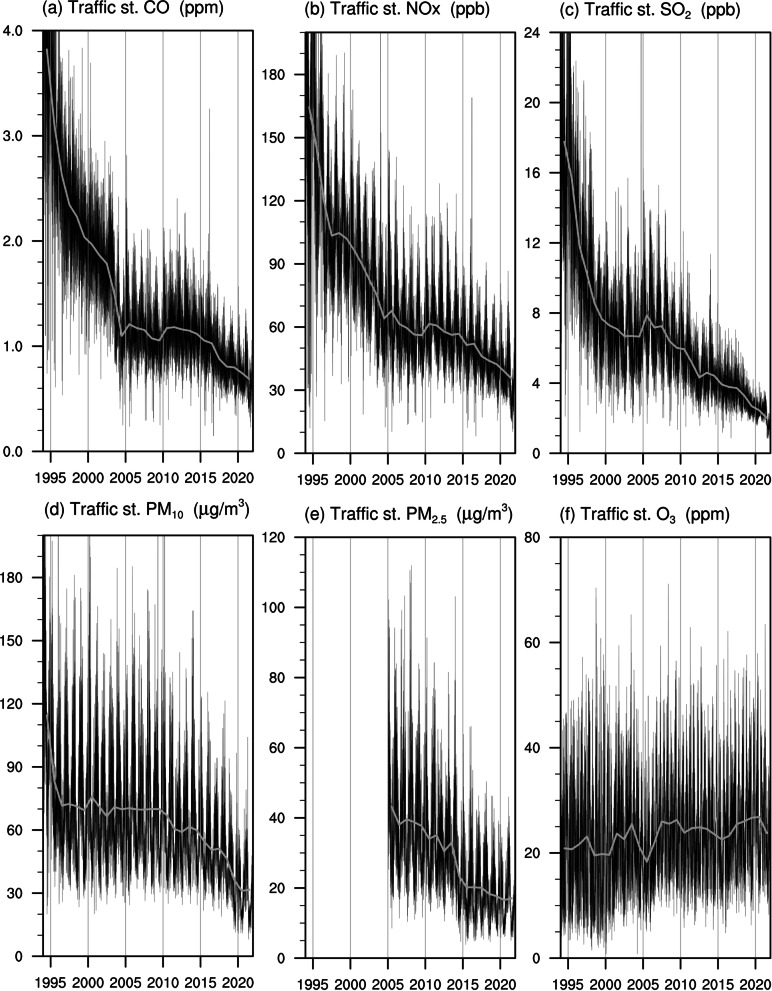
Same as Fig. 2 but monitored by the traffic stations

Similar analysis but for the air pollutants observed by all the traffic stations across Taiwan are shown in Fig. 3. Almost all kinds of air pollutants again present a decline pattern in concentration over the analyzed years despite a slight increase seen in the concentration of O_3_ during the same period. These results not only reflect the great efforts putting into air quality improvement in Taiwan, but also indicate a challenge to face in terms of the mitigation of O_3_. Generally, these two types of air quality monitoring stations recorded similar long-term tendencies in concentration for individual air pollutants, while there are detailed differences when certain air pollutants are further examined. In the traffic air quality monitoring, the main traffic-related air pollutants, namely CO and NO_x_, reveal much bigger rate of reduction over these years. It is worth a note that SO_2_ observed by the traffic stations also reveals a more significant decrease in concentration over the same years, yet road traffic is not regarded as a main source of SO_2_ by the report from the TEDS (EPA 2021a). In comparison, although PM_10_ and PM_2.5_ are also related to road traffic, their concentration values here do not largely differ from those observed by the general stations.

The results above corroborate the effectiveness of air pollution mitigation in Taiwan, and these achievements should be credited to a multi-pronged strategy for air quality improvement containing promotions of low sulphur fuel or greener facilities as well as continuously stringent emission and fuel economy regulations for automobiles (Wang et al. 2000; Tan et al. 2009, 2013; Chen et al. 2019; Tseng and Ng 2021). However, challenges to further improve air quality will raise when the concentration of an air pollutant is suppressed at a certain low level. In other words, associated strategies need to stay up to date and flexible to adapt the latest air quality condition.

Since the outbreak of COVID-19 in late 2019 many countries have implemented various measures such as quarantines or lockdowns to reduce the growth of outbreak which also brought profound impacts in many aspects. On the other hand, the epidemic offered bonus benefits to the earth with some ease in air pollution (Berman and Ebisu 2020; Gautam 2020) due to the extreme changes in human behavior caused by mandatory measures implemented to defeat the virus. From here onwards, interannual variations of the major air pollutants monitored by all the general and traffic stations across Taiwan will be shortened to a shorter 5-year period (2017–2021) in order to delve into the changes of local air quality after the outbreak of COVID-19. Moreover, a simple linear regression is adopted for estimating a linear trend for each air pollutant.

**Fig. 4:**
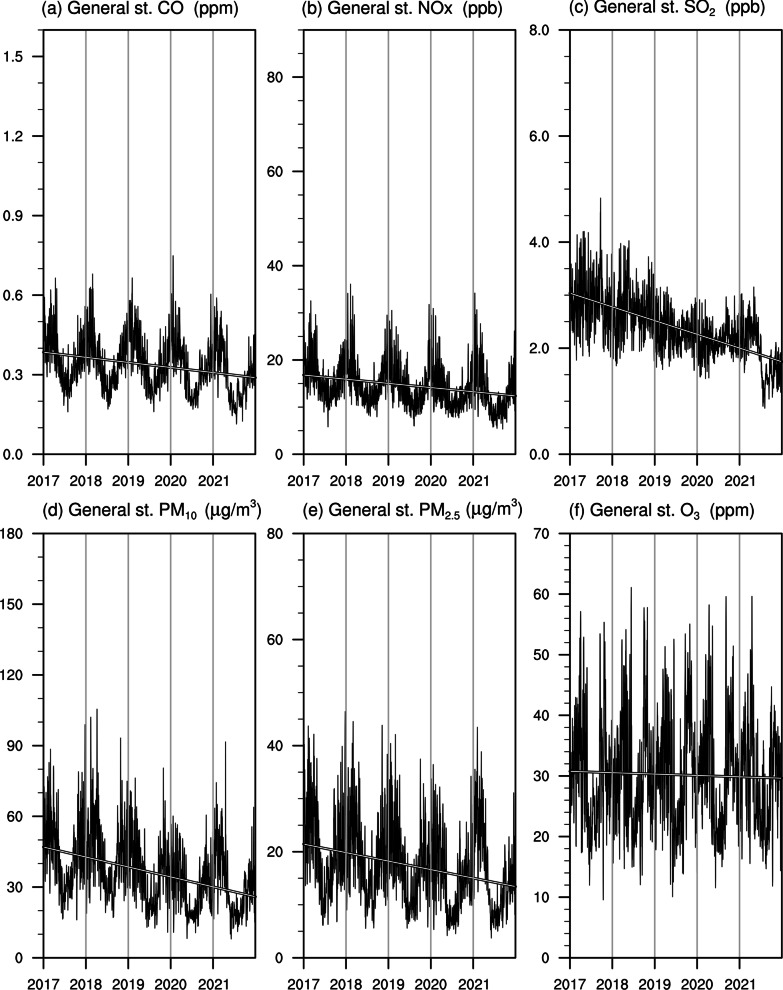
Variations of daily (**a)** CO, (**b)** NO_x_, (**c)** SO_2_, (**d)** PM_10_, (**e)** PM_2.5_, (**f)** O_3_ concentration (black line) monitored by the general stations with a trend over the years from 2017 to 2021 (gray line)

**Fig. 5:**
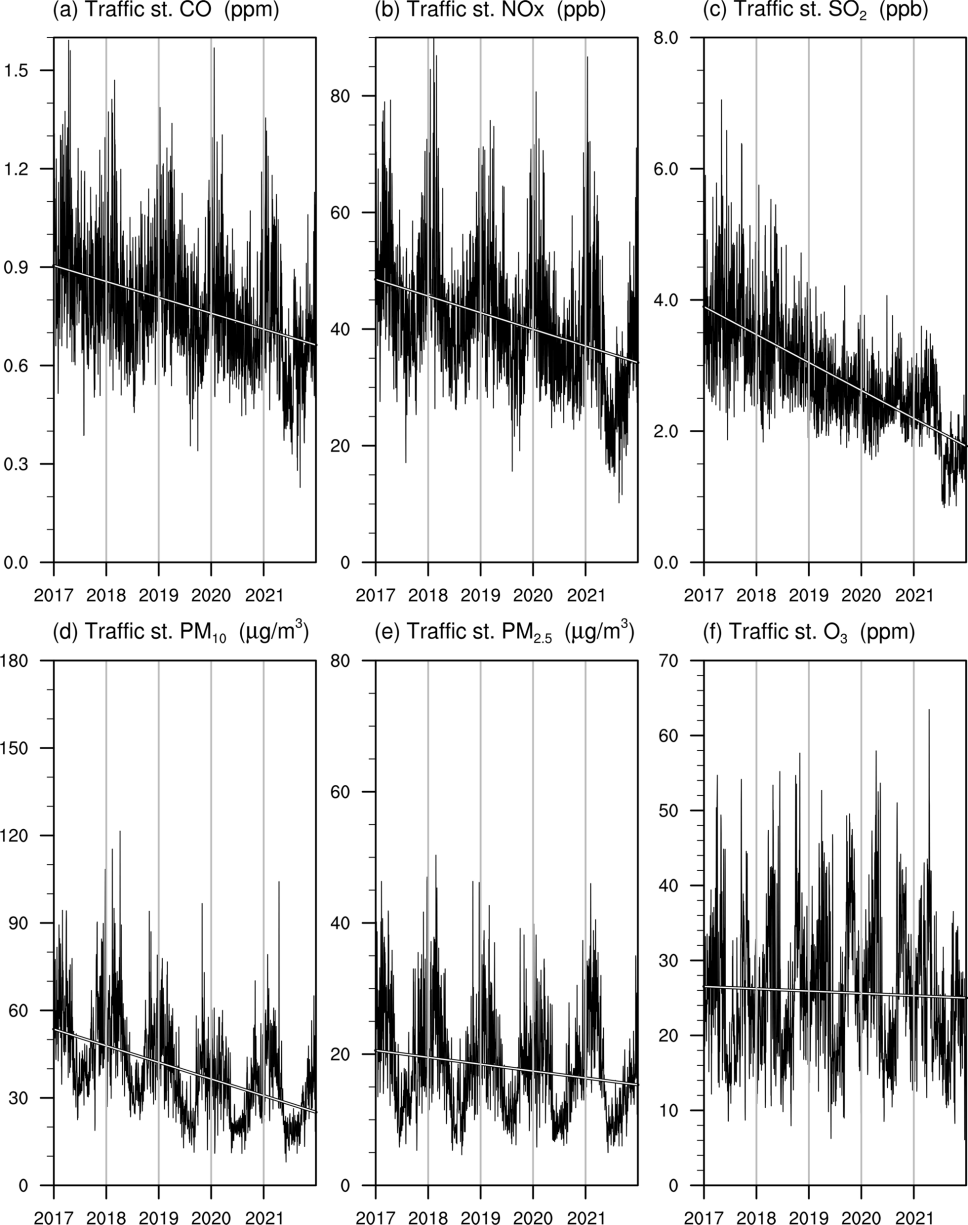
Same as Fig. 4 but monitored by the traffic stations

Most of the air pollutants observed by the general stations reveal regular annual cycle characteristics with a clear exception of SO_2_ which varies particularly unusual after the outbreak of COVID-19 since the end of 2019 (Fig. 4). Almost all of them present explicit decline trends in concentration over recent five years, while the trend of O_3_ concentration stays nearly unchanged. Similar investigations but for the pollution observed by the traffic stations are demonstrated in Fig. 5. The variations of the two traffic-related air pollutants (CO and NO_x_) together with SO_2_ again become much more fluctuant than they behave in the general air quality monitoring. Other than that, most of the air pollutants except for O_3_ indicate clear decreasing tendencies over the years which are similar to the results seen in Fig. 4. Given that all these results contain clear long-term signals, it is essential to remove long-term changes to receive real variation messages when the investigation concentrates on individual years.

## The impact of COVID-19 on air quality in Taiwan

At the early stage of COVID-19 in 2020, although the epidemic continued to spread and the world started to suffer from its ravage, Taiwan seemed to be an exception benefited by the extremely strict border controls. The risk of exposure to the epidemic remained low in Taiwan until the end of 2020 when the society became more vulnerable to the new coronavirus variants with several major cluster infections. On April 20 of 2021 a major cluster infection was identified and since then the virus rapidly spread in Northern Taiwan including Taipei City, New Taipei City and Ilan in May. The spread led to the begin of the first severe local transmitted in Taiwan and in turn, the announcement of the Level 3 alert for Taipei and New Taipei City in mid-May, followed by the nationwide Level 3 alert four days later. Unlike the strict lockdowns implemented in many countries, the Level 3 alert did not mandatorily restrict civilian movements; however, the majority of people particularly in Taipei and New Taipei City spontaneously limited their activities and worked from home during the Level 3 alert until July 28 when the alert was lowered to Level 2.

### Variations of the traffic volume during COVID-19 years

**Fig. 6:**
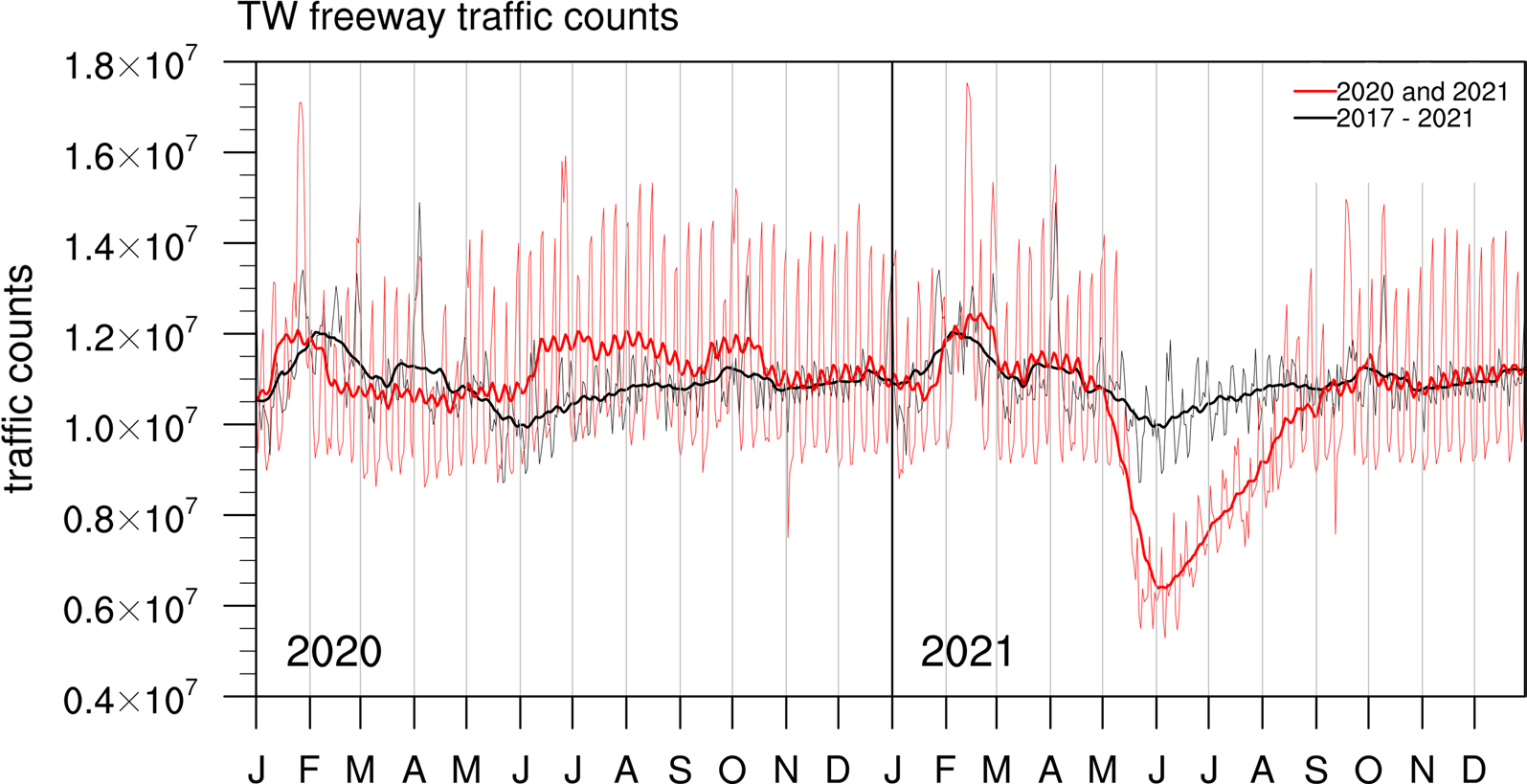
Variations of the daily freeway traffic volume collected by the ETC (thin line) on the freeways with its 31-day running mean (bold line). The 5-year mean values (2017–2021) are shown in black lines and the values in 2020–2021 are shown in red lines. Shaded areas denote the period of the Level 3 alert

During the Level 3 alert, public concern about personal health led to voluntary quarantines and therefore an abrupt decrease in daily travelling were induced. Given that fact, variations of transportation at a place should well reflect the degree of severity of the epidemic. Having examined the daily traffic volume on the north–south direction freeways in 2017–2021, this study finds the relevant result is rather related to the condition of the epidemic. Figure 6 presents the daily traffic volume through the ETC system after the outbreak of the epidemic including the Level 3 alert from mid-May to late July in 2021. The traffic volume in the early months of 2020 fell short of the mean state of recent five years in response to the concern about the initial appearance of local cases. As the severity of the epidemic stayed at an extremely low level since late April, the travelling volume significantly grew up during the summer and fall. Although it slightly decreased afterwards, the numbers were still in line with the 5-year mean values.

When the local crisis happened in late April 2021, an abrupt crash in the traffic volume since May was clearly identified. The volume reaches the bottom in early June with a nearly 40% reduction within just one month. Although the quarantine measure during the Level 3 alert was not mandatory for healthy people, the travelling volume falling off a cliff in such a short period well interprets serious public concern over the severe epidemic which was an unprecedented event in history. In early June the daily new confirmed cases still stayed at high levels; however, the traffic volume started to gradually return to usual state which occurred much earlier than the alert being lowered to Level 2 on July 28, 2021. After reaching the bottom, the traffic took more than three months back to its normal condition by early September, which is longer than we expect. This could be attributed to the mixed attitudes between serious concern and burnout among the public caused by the epidemic and associated prevention measures applied to combat the outbreak, while the relevant discussions are outside the scope of this study.

### Variations of the major air pollutants during COVID-19 years

According to the research of Tan et al. (2009), the observed “holiday effect” during the Chinese New Year and non-Chinese New Year periods in Taiwan provided evidence of the impact of human activity on ambient air quality. Given that the Level 3 alert mainly disrupted customs in daily life and the operation of companies, the relevant changes of emissions should have been recorded by the general, industrial and traffic stations which respectively represent the air quality status in environment, industrial areas and areas with heavy traffic. Therefore, this study performs detrending operations for all the studied air pollutants to remove long-term change signals, and then presents the variations of the concentrations on a daily basis in 2020–2021 with associated mean annual cycles of recent five years to investigate the change of air quality during COVID-19. In this section, all the general, traffic and industrial stations across Taiwan are analyzed to present associated ambient air quality in Taiwan, and only general stations within each studied region are considered in regional air quality investigation parts.

**Fig. 7:**
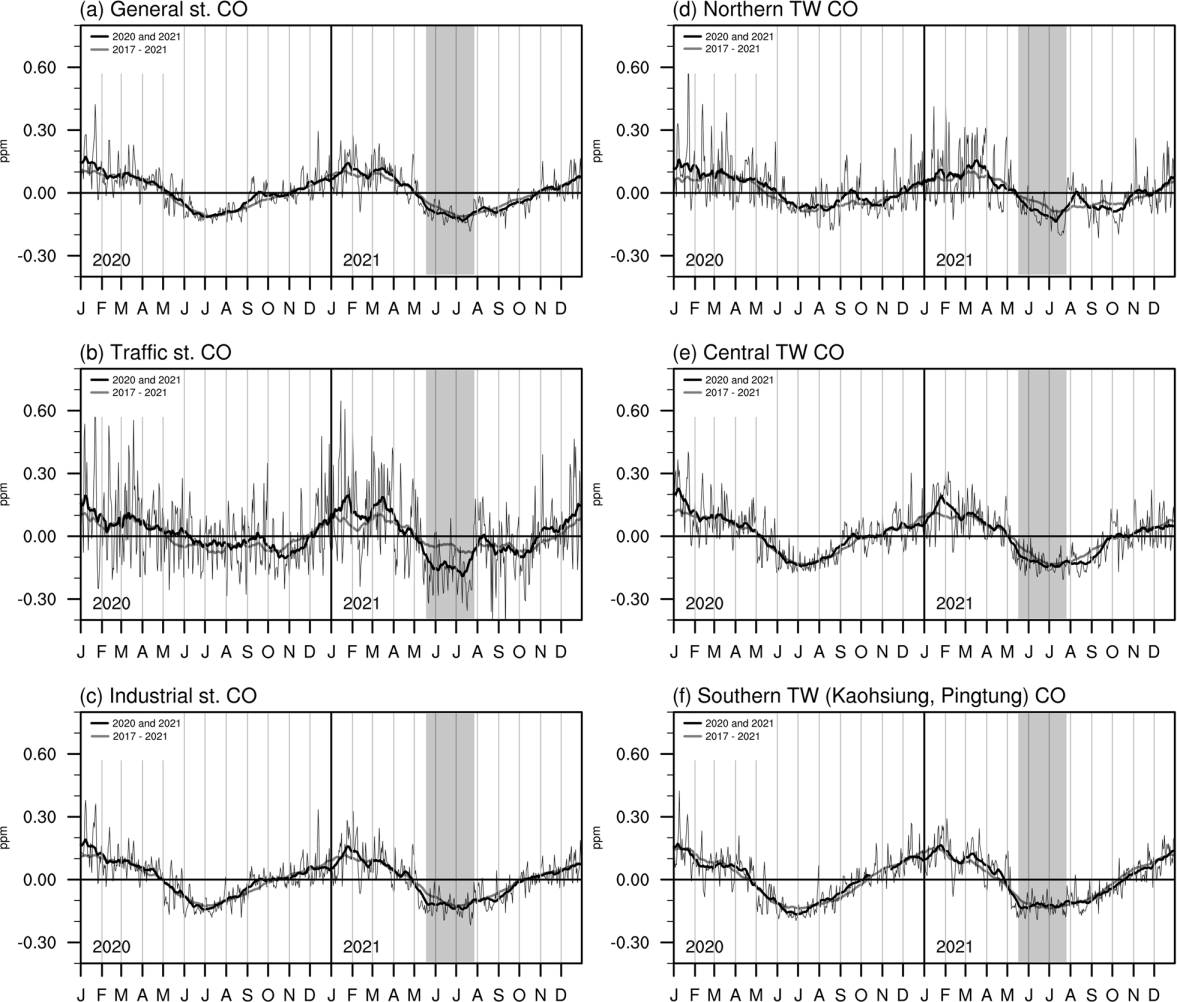
The annual cycle of CO concentration in 2020–2021 monitored by the (**a)** general stations, (**b)** traffic stations, (**c)** industrial stations, and monitored by the general stations in the (**d)** Northern Taiwan region, (**e)** Central Taiwan region, (**f)** Southern Taiwan region. The daily values are shown in thin black line with 31-day running mean in bold black line, and the 5-year mean values (2017–2021) are shown in gray line. Shaded areas denote the period of the Level 3 alert. All the variations have performed detrending operations to remove long-term change signals

The relevant investigations are shown in Fig. 7 which demonstrates the variations of CO concentration by several air quality monitoring station types and air quality regions. After the removal of long-term change signals, the variations of CO observed by the general stations in 2020–2021 mostly stay in line with the 5-year mean values (Fig. 7a), which implies the concentration of CO in populous sites was not under the influence of the epidemic. In contrast, being one of the major traffic air pollutants, CO observed by the traffic stations shows notable differences from the mean state especially during the Level 3 alert (Fig. 7b). Since the risk of exposure to the epidemic in Taiwan stayed low in 2020, CO concentration from the traffic mostly stays at the level of the 5-year mean with a slight increase from May to October. This period well matches the time with the significant growth of the travelling volume seen in Fig. 1 which is possibly attributed to the complacent altitude because of continuous zero new case. The situation of higher-than-usual CO increase carries over in early 2021 until an abnormal drop in concentration happens since May due to the first major local outbreak and the subsequent Level 3 alert. Since June the concentration of CO keeps dropping in a relatively less intense manner until it reaches the bottom in early July, and then the value quickly bounces back to the usual state after the end of the Level 3 alert in late July. Although the reduction in traffic-related emissions during that period is expected when the majority of people tended to avoid travelling if not necessary, CO concentration meets the lowest point about a month later than the bottom of the traffic volume and bounces back to the usual state about a month earlier than the restoration of the travelling. In short, the variations of CO concentration during the Level 3 alert do not concurrently respond to the associated changes in travel frequency. It implies that traffic emissions, despite being a main contributor, may not be the only factor affecting the variations of CO concentration. As for CO emitted from industrial areas, the variation characteristics in these two years do not notably depart from the 5-year mean state despite a negligible and temporal decrease right after the outbreak of the local epidemic in late April 2021 (Fig. 7c).

Although the epicenter of the epidemic in 2021 mainly concentrated in Northern Taiwan, the effect of the Level 3 alert comprises all cities and counties and hence the relevant impact on air quality in other regions of Taiwan is of interest. Hence, this study selects three air quality regions representing the three major metropolitan areas in Northern, Central and Southern Taiwan, and then examines data observed only by the general stations within each air quality region to understand relevant air quality conditions during the epidemic. In Fig. 7d–f, all the three regions show almost no difference from their usual states in 2020, while only Northern Taiwan where the epicenter of the epidemic located presents some moderate fluctuations in CO concentration after the outbreak in May 2021. In comparison with that, the relevant variations in the other two regions especially in Southern Taiwan stay in relatively usual states. However, the diversity of regional air quality conditions between the Northern Taiwan region and the other two populous sites is subtle.

**Fig. 8:**
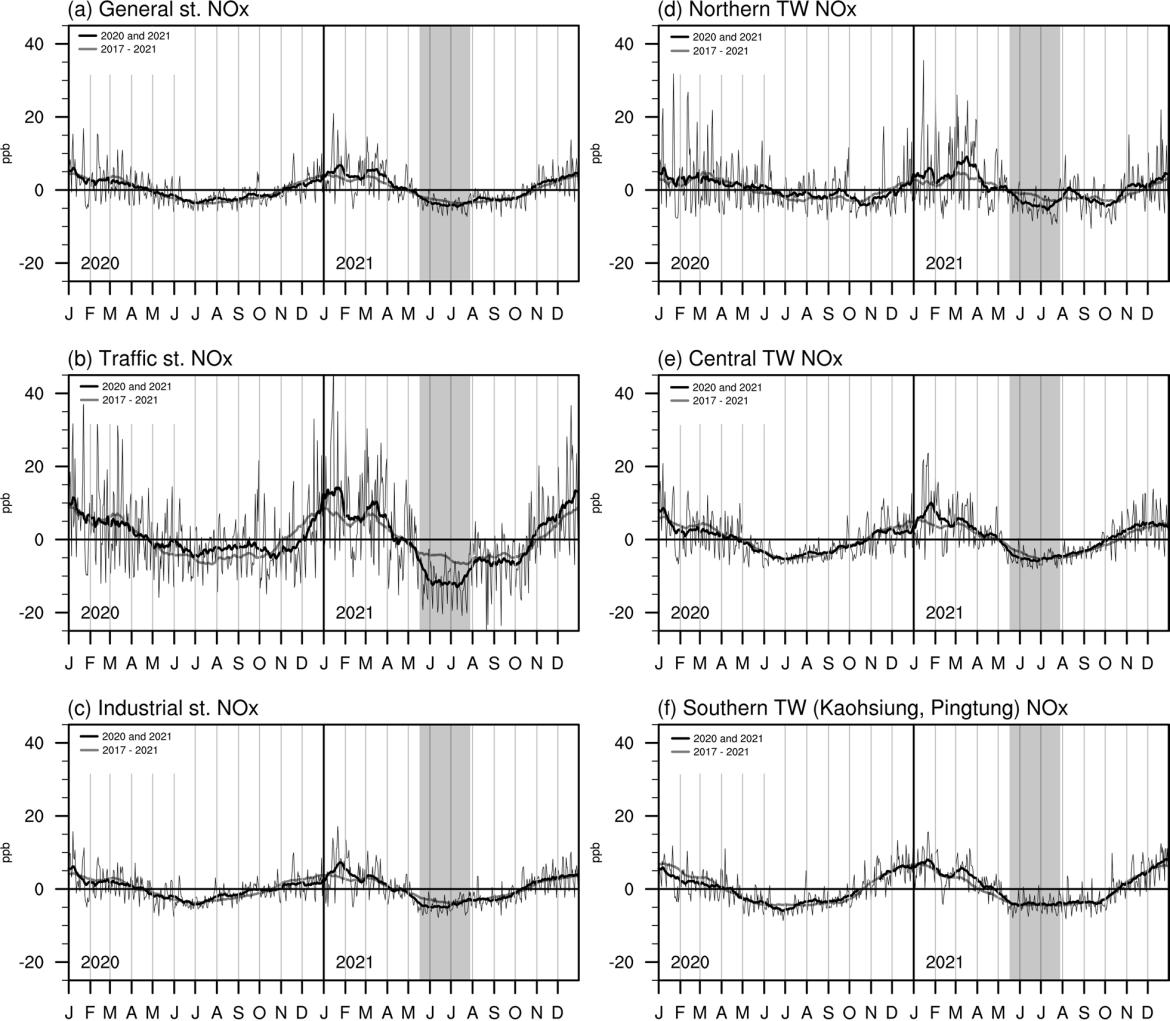
Same as Fig. 7 but for the concentration of NO_x_

Same analysis is applied to another main traffic-related air pollutant NO_x_. In Fig. 8a, NO_x_ concentration recorded by the general stations in 2020–2021 varies nearly identical to the 5-year mean state, which means the outbreak of the epidemic exert little influences on the relevant emissions in populous sites. In contrast, the traffic stations report more fluctuations in NO_x_ concentration during the same period with a slight increase from May to October in 2020 and an intense drop during the Level 3 alert in 2021 (Fig. 8b). Same as the analysis of CO concentration, the slight NO_x_ increase in 2020 should have been a consequence of the abnormal traffic increase during the low-risk period of the epidemic, and the decrease of the traffic volume due to the Level 3 alert mainly accounted for the intense NO_x_ drop in 2021. As for industrial emissions, the result of NO_x_ concentration monitored by the industrial stations varies similarly to the mean state, meaning the relevant emissions were not greatly influenced by the outbreak of the epidemic (Fig. 8c).

The analysis of NO_x_ concentration in the three selected air quality regions during the epidemic (Fig. 8d–f) reveals similar results as those of CO concentration. The variation patterns of NO_x_ in all these regions mostly stay in line with their 5-year mean states in 2020; however, some fluctuations appear in Northern Taiwan during 2021. In comparison to the Northern Taiwan region, air quality in the other two regions almost remains the same as their usual states during these two years. Even though the result in the Northern Taiwan region is not notably different from the others, it more or less reflects the fact that Northern Taiwan was at the epicenter of the epidemic during that time and regional air quality was impacted.

**Fig. 9:**
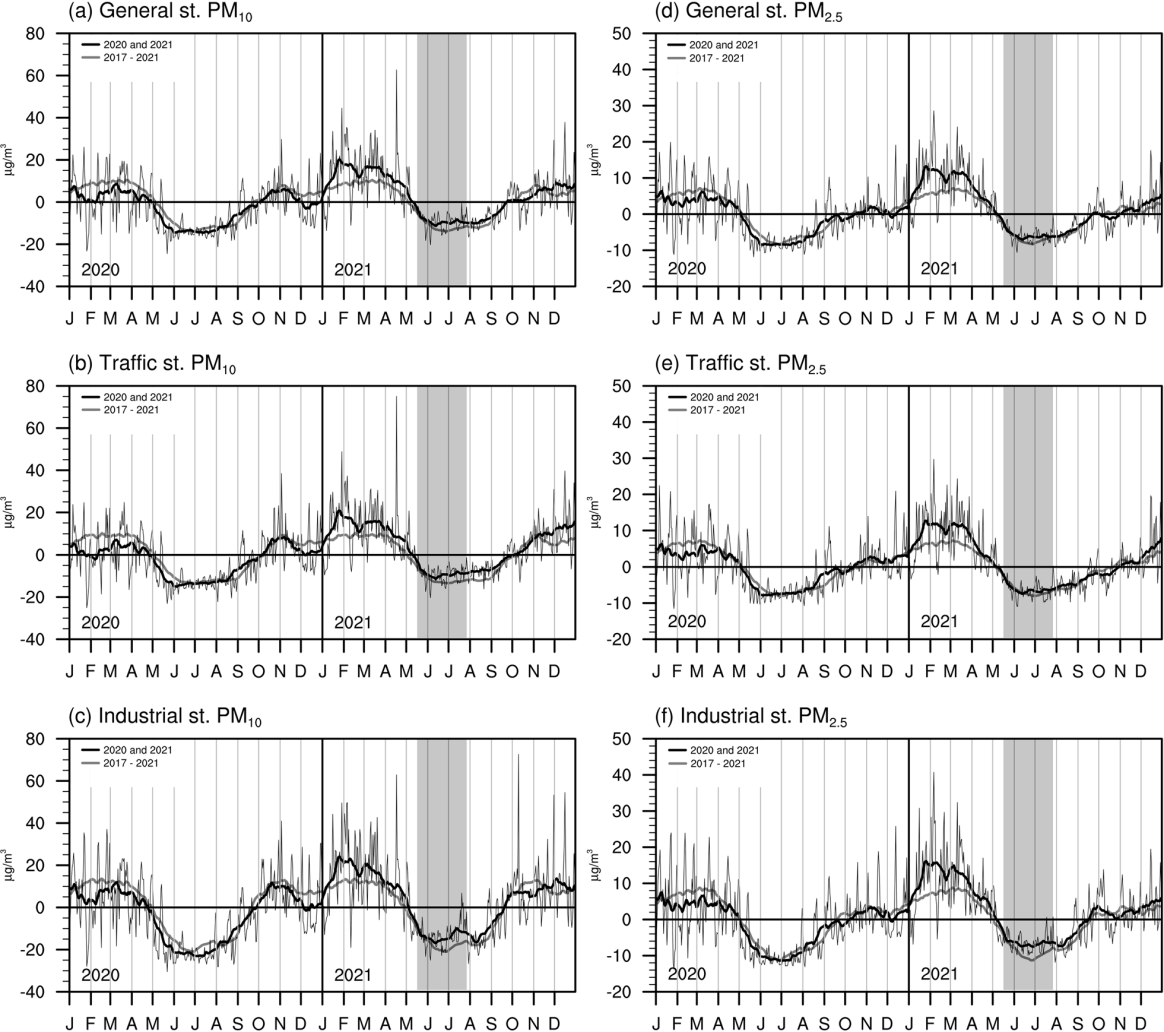
The annual cycle of PM_10_ concentration in 2020–2021 monitored by the (**a)** general stations, (**b)** traffic stations and **c** industrial stations. (**d–f)** are same as (**a–c)** but for PM_2.5_ concentration. The daily values are shown in thin black line with 31-day running mean in bold black line, and the 5-year mean values (2017–2021) are shown in gray line. Shaded areas denote the period of the Level 3 alert. All the variations have performed detrending operations to remove long-term change signals

Dust from road traffic and traffic emissions are main local sources of PM_10_ in Taiwan (EPA 2021a), while the monitored PM_10_ reveals different variation patterns as compared with CO and NO_x_ which are sensitive to the change of the traffic volume (Fig. 9a–c). All types of air quality monitoring including the traffic stations recorded smaller PM_10_ variation amplitudes during the first half of 2020 and rather normal conditions afterwards when the traffic was busy during the low-risk period of the epidemic. In the early months of 2021, PM_10_ observed by these types of stations increases faster than usual and then closes the gaps to the mean states upon the occurrence of the local epidemic and the Level 3 alert. However, the drops of PM_10_ concentrations during the local epidemic are not that intense and the values even stay slightly higher than the mean states. Considering the variety of sources for PM_10_, it is understandable that the changes of travel behavior itself during the epidemic and the Level 3 alert would not be dominant enough to hugely affect the concentrations of PM_10_.

Both NO_x_ and SO_2_ are the precursors of PM_2.5_ and the former particularly monitored by the traffic stations has shown a clear relationship with the changes of the traffic volume during the local epidemic. In contrast with that finding, the variations of PM_2.5_ observed by the general, traffic and industrial stations all present very similar patterns to the associated results of PM_10_, meaning the concentrations of PM_2.5_ only receive rather little influences from the epidemic (Fig. 9d–f). Although large decreases in NO_x_ emissions should facilitate the formation of secondary particulate matter (Huang et al. 2021), in our results, the concentration of PM_2.5_ monitored by the traffic stations (Fig. 9e) does not abruptly go up during the Level 3 alert when the concentration of traffic-related NO_x_ abnormally drops (Fig. 8b), which implies the possibility of unclear PM_2.5_ formation processes. One should also note that the patterns of the unusual increase in PM_10_ concentrations in early 2021 almost replicates in PM_2.5_ observed by all three types of stations with intense increases from January to April. These phenomena chiefly concern multiple meteorological influences including less effective washout effects and diffusion caused by abnormally dry and stable atmospheric environments throughout the first three months of 2021 (EPA 2021b). During that period, an anomalously low spring rainfall was observed which further worsened the extreme dryness on the island since the second half of 2020, leading to an unprecedented severe drought in history.

**Fig. 10:**
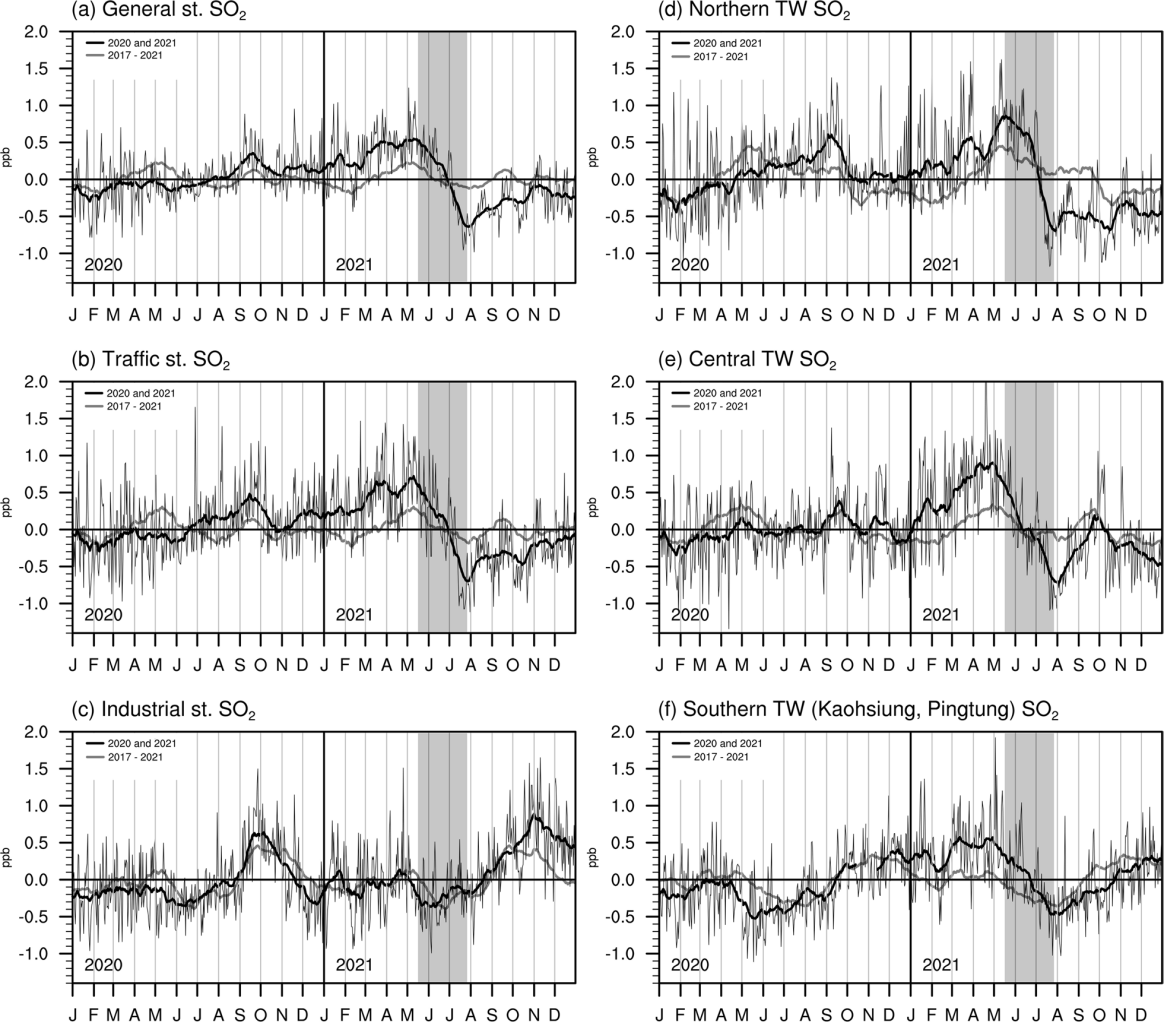
Same as Fig. 7 but for the concentration of SO_2_

The concentration of SO_2_ observed by the general stations reveals a very unusual variation pattern in 2020–2021 (Fig. 10a). Unlike the conventional two-peak pattern in a year, the concentration of SO_2_ observed by the general stations almost continuously grows since January 2020 and reaches the top by early May 2021 right before the announcement of the Level 3 alert. After the alert was raised, the concentration of SO_2_ drops much more notably than usual until reaching the bottom in late July when the Level 3 alert was lifted. This dropping pattern resembles the results found in CO and NO_x_ in terms of temporal evolution; however, diversities start to emerge in the later months of 2021. The slow SO_2_ restoration process after the end of the Level 3 alert acts very differently from the quick bounces seen in CO and NO_x_, suggesting a much longer impact from the epidemic. Through the analysis of the general SO_2_ monitoring, the entire unusual variation pattern is an implication that one should take other factors not being discussed here into consideration when the relevant causes are studied.

Similar but more fluctuating SO_2_ variations were also observed by the traffic stations (Fig. 10b), showing an unusual continuous growth particularly from late 2020 to the spring of 2021 and a long-lasting recession since the outbreak of the epidemic in 2021. Currently it is a consensus that the changes in travel behavior would not exert influences on SO_2_ monitoring because vehicle exhausts only make a small contribution to total SO_2_ emissions according to the report from the TEDS (EPA 2021a). However, through the analysis in this study, the abnormal SO_2_ variation pattern recorded by the traffic stations can be intuitively associated with the drop of the traffic volume during the Level 3 alert with a long-lasting impact through the end of 2021 from the epidemic. Certainly, there should be a possibility of undiscovered linkages between SO_2_ and traffic emissions which are accidentally exposed by the unprecedented upheaval in travel behavior during the epidemic and the speculation is worth a deep look in the future work.

One thing worth a mention is that the continuous growths in SO_2_ concentration observed by both the general and traffic stations should have been a factor of the discussed abnormal PM_2.5_ increases in early 2021 given that SO_2_ is a main precursor in the formation process of PM_2.5_. Our study suspects that the accumulated SO_2_ during that time was an ideal foundation for photochemical reaction to form more PM_2.5_, and the extreme dry and calm atmospheric environments with less effective washout effects and diffusion in early 2021 retained the existing PM_2.5_ in a certain place (EPA 2021b).

According to the report from the TEDS, power plants and industries account for the majority of SO_2_ emissions in Taiwan (EPA 2021a). Different from the unusual variations discussed above, the industrial SO_2_ emissions vary relatively close to the mean state in 2020–2021, which is an implication that the relevant emissions were not profoundly influenced by the epidemic (Fig. 10c). One should note that the second peak of SO_2_ concentration in late October 2021 grows more intensively than usual, while this abnormal growth happens few months later than the most severe time of the epidemic. For this reason, the epidemic and the following alert should not be responsible for this unusual SO_2_ increase.

The general stations recorded an unusual SO_2_ variation pattern after the outbreak of the local epidemic, hence it is of interest to explore regional differences between the three air quality regions. Since the initial outbreak of COVID-19, the Northern Taiwan region reveals a completely different SO_2_ variation pattern even during the low-risk period of the epidemic in 2020, showing a 4-month delay to reach the peak versus the usual condition (Fig. 10d). In early 2021 the concentration of SO_2_ in Northern Taiwan unusually increases and then encounters an intense drop since the announcement of the Level 3 alert in May. Although the value stops decreasing when the Level 3 alert ended in July 2021, it does not disclose any significant sign of restoration until October.

The results once again suggest clear regional disparities in terms of the impact of the epidemic when making a comparison with the Central and Southern Taiwan regions (Fig. 10e and f). Different from the unusual variation pattern in the Northern Taiwan region, the concentration of SO_2_ in Central and Southern Taiwan varies quite similarly to the usual conditions in 2020 although these regions also experience more SO_2_ increases from the end of 2020 through the spring of 2021. These two regions also encounter significant SO_2_ decreases upon the outbreak of the local epidemic in April 2021, while the extents of the decreases are relatively mild compared with Northern Taiwan and the impact from the epidemic becomes weaker towards the South. The values start to resume right after the end of the Level 3 alert; however, unlike the Northern Taiwan region, the variations of SO_2_ in these two regions quickly go back to the normal states especially in Southern Taiwan where it is far away from the epicenter of the local epidemic in 2021.

**Fig. 11:**
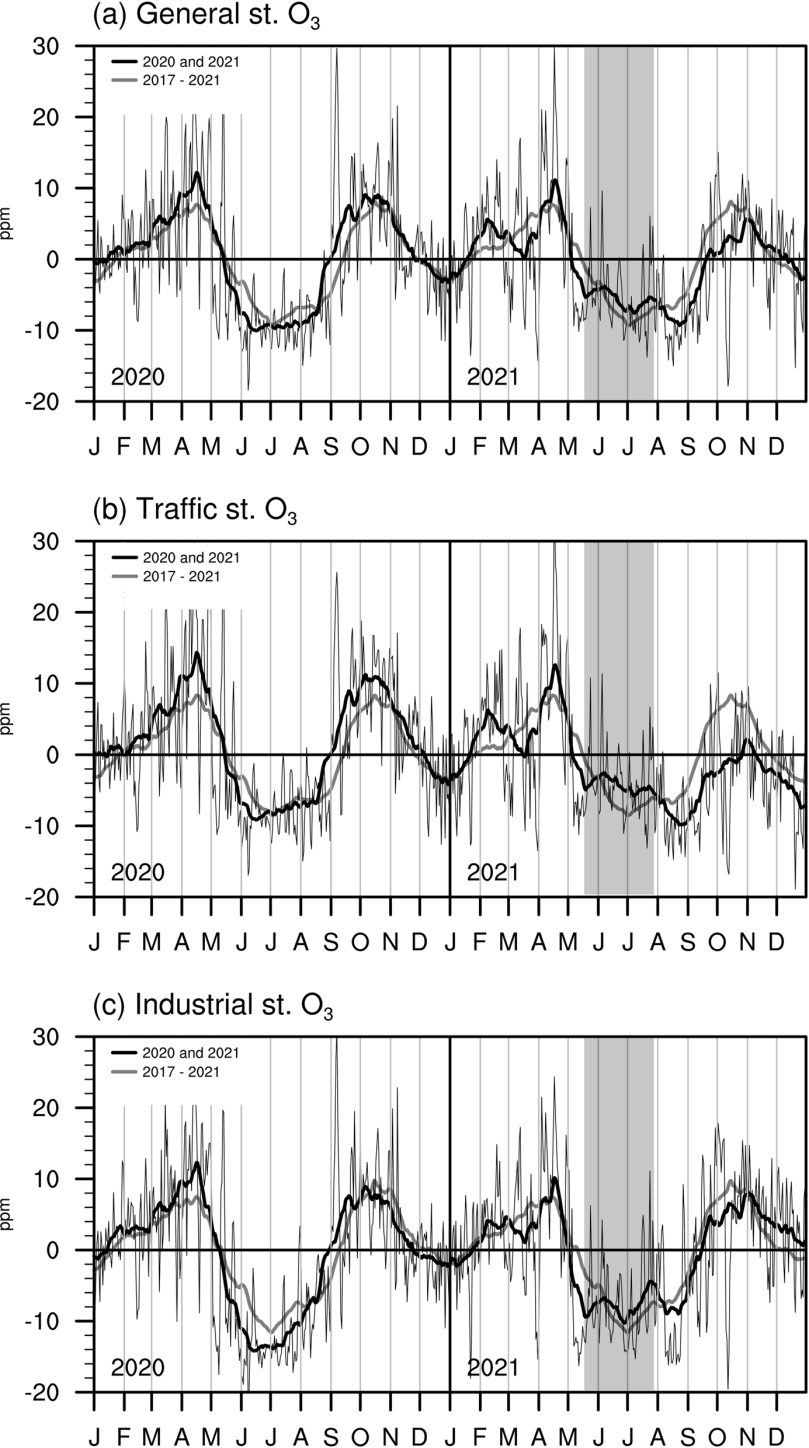
Same as Fig. 9 but for the concentration of O_3_

The concentration of O_3_ shows little variation diversities compared with the usual state regardless of the types of air quality monitoring (Fig. 11a–c). A small decrease in concentration happens right before the outbreak of the local epidemic from February to April in 2021. Since then the variation pattern somewhat returns to usual condition with more ups and downs during the severe period of the epidemic. These results do not reveal clear relationships with the changes of travel behavior even though the precursor of O_3_ (e.g. NO_x_) shows a clear indication of being influenced by the drop of traffic emissions during the Level 3 alert. This unpredictable nation of O_3_ is ascribed to its complex formation process which involves NO_x_, VOCs, ozone photochemistry process and the delicate balance between the concentrations of VOCs and NO_x_. Therefore, the simple cause-and-effect relationship between a secondary air pollutant and its associated sources is not always guaranteed, and it is impossible to reasonably assess a secondary air pollutant through only examining the change of one or two associated precursors.

## Concluding remarks

Over the past decades, a set of specific techniques and measures have been implemented in Taiwan to achieve reduction goals in air pollution including several common air pollutants such as CO, NO_x_, PM_10_, PM_2.5_, SO_2_, and O_3_. Although efforts putting into air quality improvement in Taiwan have paid off with notable long-term decreasing trends found in the concentrations of most air pollutants, the mitigation of some secondary pollutants such as O_3_ remains an extremely challenging task due to the complex formation process involving synergistic effects of precursor reductions and meteorological influences.

Among all the sources of air pollutants in Taiwan, industrial and traffic-related emissions mainly account for most of the air-polluted situations. However, since the outbreak of COVID-19 in late 2019, bustling anthropogenic activities that people across the world are used to have been comprehensively changed due to mandatory measures implemented to combat the outbreak. In contrast with the severe COVID-19 crises in most of the countries, Taiwan experienced a low-risk period of the epidemic in 2020, while a major local epidemic burst into the community in late April 2021 and triggered the announcement of the Level 3 alert from mid-May to late July. Although the alert did not mandatorily restrict public activities, the society still pressed the pause button for itself with minimal civilian activities in the daily routine during the alert, which can be clearly identified by a sudden drop in the daily ETC freeway traffic volume report.

Previous overseas cases have shown that the unprecedented changes in anthropogenic activities should provide the environment an unintended breathing space during the COVID-19 crises, while this study argues that it is not always necessarily the case in Taiwan and the result mainly depends on the type of investigated air pollutant and target air quality monitoring purpose. Among several major air pollutants in Taiwan, CO and NO_x_ are firmly associated with traffic emissions. Hence, the variations of these two air pollutants monitored by the traffic stations well resonate with the sudden drop in the freeway traffic volume records, showing an unusual decrease during the local epidemic and a restoration upon the end of the Level 3 alert. When the entire Taiwan is treated as a whole, the results of CO and NO_x_ recorded by the general stations do not reveal clear dropping signals during the same period. However, regional diversities emerge when the three studied air quality regions are individually examined. Since the epicenter of the epidemic was mainly in Taipei metropolitan, these two air pollutants in Northern Taiwan receive more impact from the epidemic than the other two regions in Central and Southern Taiwan although the differences are not clearly distinguishable.

According to the report from the TEDS, current perspective regards power plants and industries as the major sources of SO_2_ emissions, and vehicle exhausts only make a small contribution to total SO_2_ emissions (EPA 2021a). However, this study suggests different points of view with some unexpected findings. As for industrial SO_2_ emissions, there is no clear impact from the epidemic and the Level 3 alert given that the analyzed industrial SO_2_ emissions in 2021 stay rather in line with the usual state. In contrast, through the investigations into the general and traffic air quality monitoring, a clear relationship between SO_2_ concentration and the changes of travel behavior during the epidemic is shown with an intense and prolonged impact on SO_2_ concentration and more conspicuous regional differences than the results of CO and NO_x_.

As for PM_10_ and PM_2.5_, although the sources of these air pollutants are relevant to road traffic (EPA 2021a), the results clearly indicate that they are not sensitive to the changes of the traffic volume during the epidemic and the Level 3 alert. Considering the variety of sources for PM_10_ and the complexity of PM_2.5_ formation process, it is reasonable to conclude that the drop of traffic-related emissions in 2021 was not a crucial factor that directly or indirectly exerted great influences on these air pollutants. Different from any other air pollutants analyzed in this study, O_3_ does not show identified linkage to the change of anthropogenic activities during the epidemic regardless of the type of air quality monitoring. The unpredictable nation of this secondary air pollutant is attributed to its complex formation process which involves photochemistry process and the delicate balance of its precursors. For this reason, there are always challenges for scientific fields to overcome in terms of plans for O_3_ management.

Although COVID-19 brought about profound impacts in many ways, from a different point of view, the consequential unprecedented upheaval also provided a practical experience for scientists to explore new aspects in air quality which were possibly concealed from compound factors in normal times. For instance, the transmission rate of COVID-19 may be affected by air quality (Shahzad et al. 2020). Therefore, this study seizes the rare opportunity to examine relationships between individual air pollutants and the changes of travel behavior in Taiwan during the epidemic. The relevant analysis reveals both expectable and unanticipated findings and the latter suggests some new perspectives about the nation of certain air pollutants which largely divert from the current consensuses. In this regard, further re-examination into these air pollutants is recommended and hence any new possible conclusion should provide practical and valuable knowledge for upcoming air quality improvement projects specifically in the management of traffic emissions.

## Data Availability

Air quality monitoring daily data managed by the Environmental Protection Administration Environmental Information Open Platform can be downloaded at https://data.epa.gov.tw/. Traffic statistics of various types of vehicles (M03A) through Traffic Data Collection System (TDCS) managed by the Freeway Bureau of the Ministry of Transportation and Communications (MOTC) can be downloaded at https://tisvcloud.freeway.gov.tw/history/TDCS/M03A/.
